# The Use of Tick Salivary Proteins as Novel Therapeutics

**DOI:** 10.3389/fphys.2019.00812

**Published:** 2019-06-26

**Authors:** Jindřich Chmelař, Jan Kotál, Anna Kovaříková, Michail Kotsyfakis

**Affiliations:** ^1^Department of Medical Biology, Faculty of Science, University of South Bohemia in České Budějovice, České Budějovice, Czechia; ^2^Laboratory of Genomics and Proteomics of Disease Vectors, Biology Centre CAS, Institute of Parasitology, České Budějovice, Czechia

**Keywords:** ticks, therapeutics, immunomodulation, salivary proteins, anti-inflammatory proteins, hemostasis

## Abstract

The last three decades of research into tick salivary components have revealed several proteins with important pharmacological and immunological activities. Two primary interests have driven research into tick salivary secretions: the search for suitable pathogen transmission blocking or “anti-tick” vaccine candidates and the search for novel therapeutics derived from tick salivary components. Intensive basic research in the field of tick salivary gland transcriptomics and proteomics has identified several major protein families that play important roles in tick feeding and overcoming vertebrate anti-tick responses. Moreover, these families contain members with unrealized therapeutic potential. Here we review the major tick salivary protein families exploitable in medical applications such as immunomodulation, inhibition of hemostasis and inflammation. Moreover, we discuss the potential, opportunities, and challenges in searching for novel tick-derived drugs.

## Introduction

The data being generated in the high-throughput era are rapidly shifting research and development efforts toward novel and precise therapeutics that target specific pathological mechanisms and populations and diminish harmful side-effects (Paul et al., 2010; Jorgensen, 2011). Drug discovery pipelines can take a number of different forms including high-throughput screening of compound libraries or compounds similar to existing drugs, drug repurposing (Shim and Liu, 2014; Grammer and Lipsky, 2017), target-oriented molecular design, target searching using systems pharmacology approaches (Huang et al., 2014; Zhou et al., 2016), and searching for novel bioactive substances in various organisms. The latter approach is becoming increasingly attractive, as massive screening has proven to be less cost-effective than anticipated and, with rapid development in mass spectrometry, narrowing of active fractions to individual compounds is becoming easier (Wang et al., 2019). Apart from medicinal plants and/or herbal mixtures (Li and Weng, 2017), other sources – such as arthropods and parasites – have also proven to be useful (Cherniack, 2011; Ratcliffe et al., 2014; El-Tantawy, 2015). Unlike plant products that usually kill or repel herbivores and pests and have diverse effects on vital vertebrate physiology, parasitic proteins evolved to target specific vertebrate defense mechanisms. For example, the leech *Hirudo officinalis* produces the strongest known natural anti-coagulant hirudin, and hirudin and its artificial derivatives like bivalrudin are now used in clinical practice to treat coagulation disorders. Similarly, tick salivary proteins hold promise for the treatment of those processes they subvert, particularly immune-related diseases and hemostatic disorders.

## Tick Salivary Secretions and Tick-Host-Pathogen Interactions

Ticks are obligate blood-feeding ectoparasites of vertebrates including lizards, birds, and mammals (Jongejan and Uilenberg, 2004). As such, they must cope with diverse host defense mechanisms (Ribeiro and Francischetti, 2003; Francischetti et al., 2009) triggered by the bite/injury itself and the concomitant infection. The infections transmitted by ticks can be passive, such as from pathogens like poxviruses or apicomplexa present in blood on the tick hypostome or regurgitated during feeding (Tuppurainen et al., 2011; Hammer et al., 2016), and/or active when ticks are vectors for pathogens. Ticks can transmit bacteria of the genera *Borrelia*, *Anaplasma, Rickettsia, Francisella*, and others; protozoan parasites of the genus *Babesia*; and several viruses, with tick-borne encephalitis virus a major tick-transmitted viral pathogen in humans (Swanson et al., 2006; Coipan et al., 2013; Berggoetz et al., 2014; Jahfari et al., 2016; Kazimirova et al., 2017; Dehhaghi et al., 2019). Early works on tick saliva (Wikel, 1982; Ribeiro et al., 1985) showed that ticks actively modulate and/or inhibit host defense mechanisms, thus enabling the tick to complete its blood meal and facilitate pathogen transmission, as reviewed elsewhere (Francischetti et al., 2009; Wikel, 2013; Kotal et al., 2015). The latter effect has been described as saliva-assisted (originally saliva-activated) transmission (SAT) (Nuttall and Labuda, 2004, 2008), and several molecules have been described as SAT factors (Kazimirova and Stibraniova, 2013). It has been hypothesized that targeting salivary factors using a vaccine could block pathogen transmission (Neelakanta and Sultana, 2015), but despite successful vaccination and RNA interference experiments, such vaccines have yet to be clinically effective for most tick species (Neelakanta and Sultana, 2015). This is at least in part due to high functional redundancy among salivary components (Chmelar et al., 2016) or because salivary proteins are often beneficial but not indispensable for pathogen transmission (Pospisilova et al., 2019). However, from another perspective, these proteins represent a diverse and abundant library of pharmacoactive molecules with potential for medical exploitation. The functions and activities of numerous salivary components have been described and reviewed elsewhere (Chmelar et al., 2012, 2017; Kazimirova and Stibraniova, 2013; Simo et al., 2017). Here we review tick salivary protein families with potential for medical use, and in doing so we highlight that tick salivary secretions represent a unique source of novel drugs that are only just starting to be exploited and translated for clinical benefit.

## Tick Salivary Protein Families With Therapeutic Potential

**FIGURE 1:**
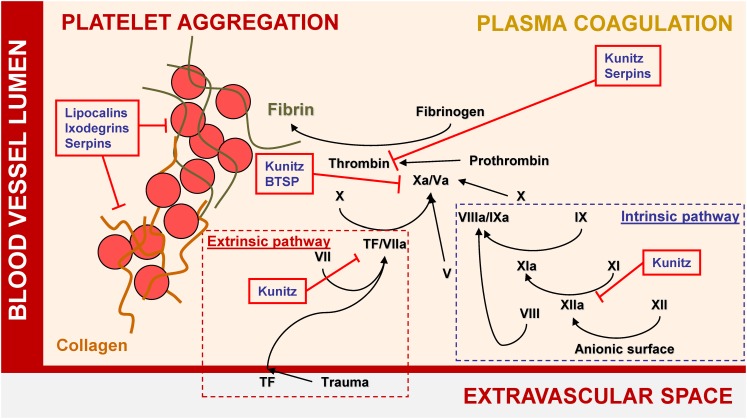
Major tick salivary protein families and their roles in hemostasis.

**FIGURE 2:**
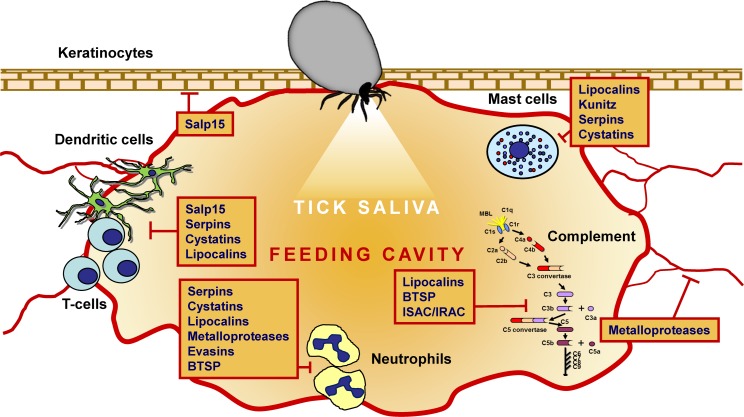
The immunomodulatory and anti-complement activities of the major tick-secreted protein families.

Thanks to comprehensive transcriptomic and proteomic studies of tick salivary glands and saliva, 10s of protein families have been identified in ticks (Oleaga et al., 2007; Oliveira et al., 2013; Schwarz et al., 2013; Mudenda et al., 2014; Tirloni et al., 2014, 2015, 2017; Karim and Ribeiro, 2015; Xu et al., 2015; Yu et al., 2015; de Castro et al., 2016; Kim et al., 2016; Esteves et al., 2017; Ribeiro et al., 2017). Some have been experimentally proven to possess anti-inflammatory, anti-hemostatic, anti-complement, and/or immunomodulatory activities, as reviewed elsewhere (Chmelar et al., 2012; Kazimirova and Stibraniova, 2013). Several diverse but abundant secreted protein groups have been repeatedly identified in transcriptomes from tick salivary glands. Their experimentally evidenced activities are summarized in Figures 1, 2, and their potential medical uses are discussed in detail below.

### Lipocalins

Lipocalins form a large family of barrel-shaped proteins with specific folds creating pockets that usually bind hydrophobic molecules such as steroids or lipids. Lipocalins form the largest and most diverse protein family in ticks. They were originally described as histamine-binding proteins (Paesen et al., 1999) but were later found to bind other biogenic amines such as serotonin (Sangamnatdej et al., 2002; Mans et al., 2008b) and bioactive lipids (leukotrienes, thromboxanes, or cholesterol) (Keller et al., 1993; Beaufays et al., 2008; Mans and Ribeiro, 2008; Roversi et al., 2017). Apart from binding small hydrophobic molecules, tick lipocalins can also bind larger proteins such as the C5 component of the complement cascade (Nunn et al., 2005; Fredslund et al., 2008). The lipocalin from the tick *Ornithodoros moubata* (Coversin) displayed a therapeutic effect in disease models (Soltys et al., 2009; Romay-Penabad et al., 2014; Pischke et al., 2017) and is already being tested in clinical trials for the treatment of thrombotic microangiopathy (Brocklebank and Kavanagh, 2017; Goodship et al., 2017). The lipocalin Japanin from *Rhipicephalus appendiculatus* was found to modulate dendritic cell differentiation, thus altering subsequent T cell-dependent cellular responses (Preston et al., 2013). Several other tick lipocalins have been successfully tested in other disease models; for example, histamine binding lipocalin Ha24 from *Hyalomma asiaticum* inhibited cell recruitment and histamine secretion in a mouse experimental asthma model (Wang et al., 2016), and rEV131 and rEV504 from *R. appendiculatus* inhibited allergic asthma and acute respiratory distress syndrome by scavenging histamine (Couillin et al., 2004; Ryffel et al., 2005; Weston-Davies et al., 2005). Therefore, tick lipocalins are proven drug candidates that target hemostasis, complement, inflammation, and acquired immunity.

### Protease Inhibitors

Endogenous protease inhibitors regulate many physiological processes in mammals, and their dysregulation leads to some serious diseases and even cancer development. Several protease inhibitor families have been identified in tick saliva. Serine protease inhibitors form four groups – Kunitz-domain inhibitors, serpins, trypsin inhibitor-like cysteine-rich domain inhibitors (TIL-domain inhibitors), and Kazal-domain inhibitors – while the cysteine protease inhibitors usually belong to the cystatin family. Tick protease inhibitors and their functions are reviewed elsewhere (Schwarz et al., 2012; Blisnick et al., 2017; Porter et al., 2017; Parizi et al., 2018), and the therapeutic potential of serpins and cystatins was outlined in our previous review (Chmelar et al., 2017). Here we discuss the therapeutic potential of the other two groups, Kunitz- and TIL-domain inhibitors.

#### Kunitz-Domain Protease Inhibitors

The Kunitz-domain protease inhibitors are the second largest family of secreted salivary proteins. Ticks possess Kunitz inhibitors with one to seven Kunitz domains, and most have been characterized as anti-coagulants that inhibit various proteases in the coagulation cascade (Corral-Rodriguez et al., 2009; Chmelar et al., 2012). Other family members possess anti-platelet activity due to integrin binding (see section “Disintegrins”). Their anti-platelet and anti-coagulatory properties make Kunitz inhibitors interesting as novel and target-specific drugs. Indeed, a Kunitz-domain protein Ir-CPI (*Ixodes ricinus* contact phase inhibitor) was shown to be a very efficient inhibitor of the contact phase of the coagulation cascade (Decrem et al., 2009), which is now being exploited in pre-clinical testing^1^. As well as hemostasis regulation, the Kunitz inhibitors Ixolaris and Amblyommin-X have displayed anti-cancer therapeutic potential (Carneiro-Lobo et al., 2009; Chudzinski-Tavassi et al., 2010; Barboza et al., 2015; de Souza et al., 2016).

Similar to lipocalins, Kunitz-domain tick inhibitors are a large and diverse group of proteins that could be used in drug development for human disease. Their highest potential lies in their anti-hemostatic properties, for which several Kunitz inhibitors have already been patented.

#### TIL-Domain Inhibitors

TIL-domain inhibitors represent another abundant group of tick salivary protease inhibitors. TIL-domain inhibitors are small (6–9 kDa), structurally conserved proteins with five disulfide bridges. Their inhibitory activity against trypsin was originally described in the parasitic nematode *Ascaris lumbricoides*. Proteins with the same cysteine scaffold were subsequently described as anti-coagulants in the helminth *Ancylostoma caninum* (Stassens et al., 1996) or in the skin secretions in the frog *Bombina bombina* (Mignogna et al., 1996). Moreover, they have also been identified in bee hemolymph (Bania et al., 1999) and as toxins in *Scorpiops jendeki* scorpion venom (Chen et al., 2013). The TIL-domain structure has been resolved, and the loop with the active P1 site is also recognized in non-tick proteins (Huang et al., 1994; Mignogna et al., 1996; Bania et al., 1999; Cierpicki et al., 2000). In addition to its anti-protease activity, scorpion TIL-domain inhibitor Sj7170 stimulated tumor growth and metastasis formation (Song et al., 2014). TIL-domain inhibitors play a regulatory role in *Drosophila* seminal fluid (Lung et al., 2002), and TIL domains are present in the large complex molecule of von Willebrand factor (Zhou et al., 2012), albeit with unknown function.

In tick salivary glands, TIL-domain inhibitors were the most abundant and diverse protein family with 108 members in *R. appendiculatus* and 64 in *I. ricinus* (Lieskovska et al., 2015; de Castro et al., 2016), suggesting that they are important in host defenses. Moreover, due to their conserved structure and known active site, TIL-domain inhibitors seem to be good candidates for protein engineering.

### Disintegrins

Disintegrins form a large group of proteins that were originally described in snake venom as 60–80 kDa cysteine-rich proteins containing an integrin-binding motif Arg-Gly-Asp (RGD). This domain is also typical in extracellular matrix proteins and enables platelet binding via integrins and subsequent thrombus formation. RGD domains in soluble proteins such as disintegrins prevent platelet aggregation, which is crucial for the success of hematophagous parasites (Assumpcao et al., 2012). Three Kunitz domain-containing disintegrins have been functionally characterized: disagregin from *O. moubata*, which shows low similarity to Kunitz inhibitors and does not actually contain an RGD domain (Karczewski et al., 1994; Karczewski and Connolly, 1997); savignygrin from *Ornithodoros savignyi* (Mans et al., 2002a,b); and monogrins from *Argas monolakensis* (Mans et al., 2008a). Another group of tick disintegrins includes variabilin (Wang et al., 1996), which was the only group member characterized and later named ixodegrins (Ixodida + disintegrins) after their discovery in the *Ixodes pacificus* transcriptome (Francischetti et al., 2005). Variabilin was shown to block the binding of fibrinogen to integrin αIIbβ3 (Wang et al., 1996). The second tick protein characterized in the ixodegrin family was YY-39 from *Ixodes scapularis*, which was shown to bind integrin αIIbβ3 and thus compete with fibrinogen binding to platelets (Tang et al., 2015). Ixodegrins are structurally similar to snake venom disintegrins and usually contain RGD or KGD domains. Given their properties, they may be candidate therapeutics as novel anti-thrombotic agents.

### Basic Tail Secreted Proteins (BTSPs)

The first BTSP family member was identified and described as one of 14 immunodominant antigens in *I. scapularis* and named Salp14 (Das et al., 2001). More homologs were subsequently found in the transcriptomes of *I. scapularis* (Valenzuela et al., 2002) and other tick species, where BTSP usually forms one of the most abundant and diverse putatively secreted groups (Francischetti et al., 2005; Schwarz et al., 2013; Karim and Ribeiro, 2015; de Castro et al., 2016). Salp14 and its smaller homolog Salp9Pac are anti-coagulants important for tick feeding, as evidenced by RNA interference studies in ticks (Narasimhan et al., 2002, 2004). Another BTSP family member, Ixonnexin, promoted fibrinolysis by accelerating plasminogen activation and inhibiting factor Xa (Assumpcao et al., 2018). An immunogen originally named P8 (Schuijt et al., 2011b) displayed lectin binding properties and acted as a complement inhibitor (Schuijt et al., 2011a), thus protecting *Borrelia* spirochetes from complement attack (Wagemakers et al., 2016).

The BTSP family contains 100s of members, some with modifications such as acidic tails or no tail instead of basic tails, which are usually composed of lysine and arginine residues (Francischetti et al., 2008). They work as anti-coagulants and specific complement inhibitors, both of which are very attractive drug targets.

### Salp15

Wikel (1982) observed that tick infestation inhibited mitogen-induced guinea-pig T cell proliferation. Since, then, many effects of tick saliva on T cell function have been described (Kotal et al., 2015). The best characterized tick salivary protein with a direct effect on T cells is Salp15 from *I. scapularis* (Anguita et al., 2002), a member of a large family of heavily glycosylated proteins that seem to be unique to prostriate ticks such as *Ixodes* spp. (Wang et al., 2014). Salp15 binds specifically to the CD4 co-receptor on T cells, thus inhibiting cell signaling, reducing IL-2 production, and promoting CD4^+^ T cell activation (Anguita et al., 2002; Garg et al., 2006; Juncadella et al., 2007). The immunomodulatory potential of Salp15 has been tested in several *in vivo* models with promising therapeutic results, e.g., in inhibiting HIV infection (Juncadella et al., 2008), experimental autoimmune encephalomyelitis (Juncadella et al., 2010), transplantation rejection (Tomas-Cortazar et al., 2017), and asthma (Paveglio et al., 2007). In addition to its anti-CD4^+^ T cell effect, Salp15 is also a suppressor of dendritic cell function (Hovius et al., 2008; den Dunnen et al., 2009), suggesting another use in targeted therapy. In addition, Salp15 inhibited TLR2-dependent inflammation and production of antimicrobial peptides by keratinocytes (Marchal et al., 2011). Apart from its immunomodulatory activities (Juncadella and Anguita, 2009), it has also been associated with protection of *Borrelia* spirochetes from the immune system (Ramamoorthi et al., 2005; Dai et al., 2009).

### Metalloproteases

Metalloproteases form large group of physiologically important enzymes that play a role in extracellular matrix degradation, protein shedding from the cell surface, and enzyme activation. Tick salivary metalloproteases accelerate fibrinolysis (Francischetti et al., 2003; Beaufays et al., 2008), thus their function is anti-hemostatic. Two proteins from *I. scapularis* from the ADAMTS family (a disintegrin and metalloprotease with thrombospondin motifs) significantly inhibited neutrophil function, broadening the range of possible metalloprotease activities (Guo et al., 2009). The abundancy and diversity of metalloproteases in tick saliva is high; however, using them as therapeutics can be problematic due to their stability and the long-term maintenance of their activity.

### Evasins

Chemokines play a role in cell recruitment to inflammation sites and therefore play crucial roles in promoting inflammatory responses (Charo and Ransohoff, 2006). Evasins form a family of chemokine-binding proteins first identified in *Rhipicephalus sanguineus* (Frauenschuh et al., 2007; Deruaz et al., 2008). Homologs were also found in *Amblyomma* and *Ixodes* species; however, in *Ixodes*, the homology is very weak (Hayward et al., 2017). Evasin-1 and evasin-4 bind CC chemokine members, while evasin-3 is specific for CXC chemokines (Frauenschuh et al., 2007; Deruaz et al., 2008). Evasin-1 can inhibit neutrophil, T cell, and macrophage migration and the production of inflammatory cytokines, from which originates its therapeutic potential. Evasin-1 has shown benefit in the treatment of pulmonary fibrosis (Russo et al., 2011) and graft versus host disease (Castor et al., 2010). Evasin-4 can interact with at least 18 CC chemokines, inhibit eosinophil recruitment (Vieira et al., 2009; Deruaz et al., 2013), and was effective against post-infarction myocardial injury and remodeling (Braunersreuther et al., 2013). Evasin-3 effectively inhibited myocardial reperfusion (Montecucco et al., 2010) and neutrophil recruitment (Deruaz et al., 2008), thereby reducing atherosclerotic vulnerability for ischemic stroke (Copin et al., 2013). Evasin-3 was also shown to inhibit neutrophil-mediated inflammation in a mouse acute pancreatitis model (Montecucco et al., 2014). The therapeutic potential of evasins is reviewed in Bonvin et al. (2016).

## Tick Proteins and Bioengineering

The preceding discussion highlights that tick salivary proteins can target every possible immune mechanism and therefore have the potential to be used as drugs, especially against disorders of hemostasis, immune-mediated inflammatory diseases, and also against tumor growth. Indeed, many tick molecules have been patented for these reasons (Nuttall and Paesen, 2000; Nunn, 2004; Chudzinski-Tavassi et al., 2005); moreover, Coversin has already been, and continues to be, tested in clinical trials (ClinicalTrials.gov: NCT03829449, NCT03427060, NCT02591862, NCT03588026) for paroxysmal nocturnal hemoglobinuria and microscopic and thrombotic microangiopathy (Brocklebank and Kavanagh, 2017; Goodship et al., 2017). However, the pharmacokinetics and pharmacodynamics of tick proteins can limit their therapeutic potential. Similar to any large molecule of exogenous origin, tick salivary proteins display strong immunogenicity. This can be overcome using several modifications such as PEGylation [attachment of polyethylene glycol (PEG) polymers], which should, in addition to decreasing immunogenicity, improve solubility (Veronese and Mero, 2008; Jokerst et al., 2011). Another possibility is humanization of the proteins, in which potential T cell epitopes are eliminated or changed. This approach has been used with great success for monoclonal antibodies designed predominantly for cancer treatment by the fusion of the hypervariable parts of mouse-derived monoclonal antibodies to human heavy chains (De Groot and Scott, 2007). A similar approach could be used for tick serpins, where the reactive center loop (RCL), which is responsible for serpin specificity, would be of tick origin, and the conserved serpin scaffold would be of human origin (Silverman et al., 2010; Whisstock et al., 2010). The concept of creating novel serpins by combining the RCL and scaffold from different serpins has been proven with the fusion of the furin inhibitor B8 and the mutant variant of α1-antitrypsin (Izaguirre et al., 2013, 2019). Furthermore, a novel extracellular inhibitor of human granzyme B was produced by combining mouse RCL and a human scaffold into a single chimera (Marcet-Palacios et al., 2015). Despite these promising results, there is no experimental evidence that the same approach would be successful for tick salivary molecules as well. Our understanding of the interaction between tick salivary secretion and host immune system is still incomplete, and immune tolerance is not the only obstacle in turning tick proteins into drugs.

In addition to immunogenicity, small proteins and peptides, which many tick salivary proteins are, usually have a short circulatory half-life. This problem can be solved by fusing the studied protein with the Fc fragment of human IgG. Such a fusion was performed with evasin-4 but without success (Bonvin et al., 2016). PASylation – protein conjugation with the polymeric sequence of Pro, Ala, and Ser – is a relatively new method similar to PEGylation that can prolong protein half-life in the circulation by several days (Schlapschy et al., 2013). There are ways to modify tick salivary proteins to improve their pharmacodynamics and pharmacokinetics, some of which have already been successfully used, such as PASylation of Coversin (see section “Lipocalins”), which inhibited its degradation in plasma (Kuhn et al., 2016).

## Concluding Remarks

Based on experimental evidence and/or the membership of proteins to well-described families, the function of some tick proteins has been proven or at least inferred. For some families, functional knowledge is still lacking. Therefore, tick salivary proteins remain rather poorly explored with respect to their therapeutic applications. Most efforts aiming to develop new drugs from ticks focus on the two largest families, Kunitz-domain inhibitors and lipocalins, as these groups contain new members with novel functions via several genetic mechanisms (Mans et al., 2008b; Schwarz et al., 2014). However, other groups deserve attention as well, such as TIL-domain protease inhibitors, Salp15, evasins, and many others. This minireview summarizes the main protein groups to emphasize the therapeutic potential of tick salivary proteins and some of the problems faced with clinical translation.

## Author Contributions

JC, JK, and AK wrote the manuscript. JC prepared the figures. MK reviewed and edited the manuscript.

## Conflict of Interest Statement

The authors declare that the research was conducted in the absence of any commercial or financial relationships that could be construed as a potential conflict of interest.

## Footnotes

1 www.bioxodes.com
